# Personal and institutional factors as determinants of civic engagement among university students

**DOI:** 10.3389/fpsyg.2025.1517319

**Published:** 2025-12-05

**Authors:** Yang Lin, Danabekova Aigerim, Saba Ghayas, Misbah Malik, Sadia Niazi

**Affiliations:** 1 Faculty of Humanities and Foreign Languages, Xi’an University of Technology, Shaanxi, China; 2 School of International Languages, Shaanxi Normal University, Xi’an, China; 3 Department of Psychology, University of Sargodha, Sargodha, Pakistan

**Keywords:** institutional efficacy, student control, advocacy, direct action, organizational participation, civic engagement

## Abstract

**Background:**

The study evaluated the civic engagement model within a specific Pakistani cultural context. This study aims to examine the personal and institutional factors, contributing to civic engagement behaviors.

**Method:**

The purposive sample consisted of 307 university students aged between 18 and 26 years from the University of Sargodha. The reliability of all the scales used in this study was above 0.70.

**Results:**

The results supported the proposed model, indicating that institutional efficacy and sense of student control significantly contribute to explaining civic engagement that includes advocacy, direct action, and organizational participation. The results provide valuable insights into the relationships among attitudinal factors, civic engagement behaviors, and the influence of gender within our cultural context. The strength of the association between variables is shown by the standardized path coefficients (*β*). Results revealed that advocacy plays a role as mediator in the relationship between institutional efficacy and direct action. The study found a significant positive path coefficient (*β*) between student control and advocacy, indicating that individuals with a sense of control are more likely to engage in civic participation. Additionally, student control predicted organizational participation, leading students to actively participate in student organizations. Gender differences in the study demonstrate that males have a higher positive indirect effect of institutional efficacy on direct action through advocacy than females, and the positive direct effect of advocacy on organizational membership is more prominent for males.

**Conclusion:**

The study found that institutional efficacy and student control significantly influence advocacy, direct action, and organizational participation that define civic engagement, thus supporting the existing civic engagement model. The study extended the civic engagement model to incorporate mediation effects and investigated it in a specific cultural setting, which can be very useful for educators, lawmakers, and community organizers.

## Introduction

Civic engagement encompasses a wide range of actions and attitudes toward social and political participation that collaborate to improve the health of a democratic society (Banyan, 2023). Civic engagement alludes to people’s entanglement and active involvement in their communities and society, particularly in actions that promote the general welfare (Zaff et al., 2011). It incorporates a broad variety of activities, from campaigning and volunteering to direct political action and organizational participation (Nishishiba et al., 2005). Within this broad framework, youth civic engagement emerges as particularly significant, as young people contribute fresh perspectives, energy, and innovation to social and political processes, helping drive progress in a rapidly changing world (Shah and Khan, 2023; Earl et al., 2017). It gives a variety of prospects to advance skills and knowledge, promoting demonstrative decision-making (CIRCLE, 2022). It also leads to jazzed-up academic achievement (Rochford and Hock, 2022), better emotional well-being (Fenn et al., 2021), and can enhance both individual (Altares et al., 2022) and community advancement (Ohmer et al., 2022). Young generation is less likely to participate in traditional civic activities, but they are more likely to interact with issues connected to lifestyle values, which include moral concerns and environmental quality (Henn et al., 2022; Ahrari et al., 2015). This shifting trend of civic involvement highlights the importance of education in bridging the gap between traditional and modern modes of engagement. Education also serves as a tool to help students understand civic society. Higher education has a tremendous impact on increasing student participation in public life. Previous research has shown that higher education not only produces positive educational outcomes but also cultivates positive self-concept, improves problem-solving skills, fosters leadership development, promotes cultural awareness, and results in a high level of civic engagement among students (Subramaniam, 2019). Palmer et al. (2010) claim that higher education, as a critical component in defining democracy’s future, can develop students’ emotional, interpersonal, and ethical skills, transforming them into responsible citizens. Additionally, Prior studies have also found that university and college experiences, such as curriculum and co-curricular diversity (Garcia and Cuellar, 2018), peer interaction (Tamam and Krauss, 2014), and campus climate (Mitchell et al., 2016), improve students’ civic outcomes, moral development, and social responsibility. Research has also shown that citizens who feel empowered by their societies and are provided platforms for active participation tend to be more civically engaged, contributing to advocacy, direct action, and organizational participation (Nishishiba et al., 2005).

The civic engagement model proposed by Nishishiba et al. (2005) forms the foundation of this study. According to this model, varied factors of civic engagement behavior, such as advocacy, direct action, and organizational participation, are analytically linked to attitudinal factors that include community efficacy and a sense of citizen control. In order to understand the basic mechanisms of this civic engagement model, which reveals that attitudinal factors such as community efficacy and citizen control forecast civic engagement behaviors such as advocacy, direct action, and organizational participation, it is vital to consider the role of attitude in determining behavior. It is proposed that attitude be interpreted as a behavioral predisposition (Cherry, 2023). It can be said when an individual’s attitude toward an object or situation is triggered, it determines the conduct they adopt in response to that object or scenario. This relationship between attitude and behavior has important implications for the development of the civic engagement model.

This civic engagement model explains the development of civic engagement in students. This model states that civic self-efficacy is a crucial factor in the development of civic engagement. The idea of civic self-efficacy is taken from the self-efficacy theory of Bandura (1997). It states that an individual’s belief in their ability significantly influences civic and political engagement processes. The model suggests that students who are confident in their civic capacities are more likely to participate in community or political activities. This confidence can be developed through communal modeling and supportive circumstances that foster civic participation (Bandura, 1997).

Moreover, some aspects of the civic engagement model are based on the postulates of Bronfenbrenner’s Ecological Systems Theory, which highlights how different environmental systems affect human development, influencing the model (Colby et al., 2003). The study highlights that institutional environments influence civic attitudes and behaviors, specifically at colleges and universities. Students’ civic responsibility is seen to be enhanced by educational institutions that support inclusive participation, democratic engagement, and service-learning opportunities (Eyler and Giles, 1999).

The civic engagement model is also influenced by transformative learning theory, which suggests that significant learning experiences, together with reflective discussion, community participation, and exposure to varied perspectives, can result in changes in identity and value systems (Astin and Sax, 1998). These involvements not only provide students with knowledge but also reinforce their commitment to civic principles.

Lastly, the model integrates structural and motivational components from broader civic engagement theories. It examines how institutional support, external opportunities, and internal incentives like ethical responsibility and social concern influence civic behavior (Longley, 2022). The combination of these psychological, developmental, and structural aspects creates a comprehensive framework for understanding how civic engagement is encouraged in academic settings.

The model has been reformed to better fit university students by altering the indigenous attitude factors, which were community effectiveness and citizen control to institutional efficacy and student control, respectively. Community efficacy is the certainty of people in their capability to effect change in their communities (Nishishiba et al., 2005), while institutional efficacy is the perception of students’ impact within institutional settings, such as universities. Whereas student control emphasizes the degree to which students feel empowered to influence policies and decisions in their academic settings (university), such as involvement in academic discussions and impact on administrative decisions. In addition to attitudinal factors the behavioral facets of civic engagement, which include activities such as advocacy, direct action, and organizational participation, have been modified to adapt to the academic environment.

### Advocacy

Students exhibit their political involvement by advocacy (Longley, 2022), which is marked with interaction with peers and institutions about their ideas and concerns. This includes organizing campaigns, having discussions, and forming petitions (Mosley et al., 2020). It affects how verdicts are made and fosters a sense of liability and self-determination that boosts active partaking in improving society.

### Direct action

Participating in direct action surpasses expressing concerns in writing to public administrators or the media. It covers taking concrete steps to bring about social change, such as actively participating in rallies or rallies to express one’s ideas and opinions. Boycotts are another form of direct action in which people decisively withhold from supporting specific activities or products in order to protest or show discontent (Nishishiba et al., 2005). Civic involvement can also involve taking direct action, such as protesting, demonstrating, or boycotting, to effect social change. Furthermore, students participate in civic and social activities by actively joining organizations and associations, which Longley (2022) considers to be a sort of civic engagement.

### Organizational participation

According to Keeter et al. (2022), involvement in groups and associations demonstrates civic engagement. Organizational participation is a technique for local residents to build social capital and shape their community to reflect common values. It brings together people who have the same goal or cause, encouraging collaborative efforts to effect positive change in both the community and its local government (Zencity, 2023).

The amendments were applied to ensure that the model is applicable to university students, whose degree of civic engagement is often closely associated with their academic environment. Focus on institutional efficacy and student control allows students to navigate their academic environments to bring change in their civic participation. Similarly, adapting the behavioral components of civic engagement confirms that the model accurately represents the specific types of involvement that are dominant among students, so providing a more precise illustration of how civic engagement is demonstrated in the university setting.

The original model proposed by Nishishiba et al. (2005) was formulated in an individualistic society, current study intended to test the model in collectivistic culture, in order to examine distinct cultural differences. The main difference between individualism and collectivism is how people perceive themselves in relation to others. In a collectivist society, the self is dependent on members of the group and views group issues (such as the harmony of the group and cohesiveness) above personal ones; in contrast, in an individualist society, the self is sovereign and independent of the group; hence, individual benefits take priority over group ones (Triandis, 2018; Oyserman et al., 2002; Oyserman and Lee, 2008). Moreover, Civic involvement is shaped by deeply held cultural, social, and religious beliefs (Singh, 2023).

Furthermore, gender differences are also investigated in this study. Pakistan is a nation distinguished by prominent gender discrimination, which thus significantly influences civic involvement patterns across genders (Ali et al., 2022). Particularly women experience more hindrances to engage in public life because of systematic discrepancies, cultural values, and societal expectations (Ali et al., 2022; Firdoos et al., 2023; Mahmood, 2012). This study aims to explore how these gender differences affect students’ civic engagement. Including gender as a fundamental element of the model helps us to clarify how institutional and cultural elements interact to strengthen or hinder civic participation across gender.

This study aims to validate the civic engagement model among Pakistani students and investigate the role of institutional and personal factors in its development across different cultural contexts. Model testing is essential for generalizing beyond data, comparing in-sample and out-of-sample performance, and evaluating prediction performance while avoiding overfitting (Rocca and Yarkoni, 2021).

The original civic engagement model by Nishishiba et al. (2005) was developed using white university students in the United States, but it is crucial to test its applicability in non-Western, collectivistic cultures like Pakistan. Collectivist cultures prioritize social connectivity, interdependence, and in-group aspirations, whereas individualistic cultures prioritize self-reliance, independence, and personal ambitions (Cortina et al., 2017). Cross-cultural validation allows us to explore how institutional efficacy and student control operate in different cultural frameworks and how these factors influence civic engagement behaviors in Pakistani students.

Gender differences in Pakistan are another significant reason for testing the model. In Pakistan, where gender discrimination is prevalent (Ali et al., 2022). Studying these gender dynamics in a Pakistani context could yield different outcomes when compared to more gender-equal societies, offering a nuanced understanding of how gender shapes civic engagement.

The present study seeks to extend the model by examining whether advocacy mediates the relationship between institutional efficacy and direct action or organizational participation. Investigating mediation effects adds complexity to the model, helping to better understand the mechanisms through which institutional efficacy and student control influence civic engagement. Identifying these indirect pathways can provide deeper insights into how institutional support and students’ sense of control contribute to different types of civic engagement, highlighting areas where universities and policymakers can intervene to promote greater student involvement in civic activities.

### Hypothesis

*H1*: Institutional efficacy would be a significant predictor of advocacy, direct action, and organizational participation.

*H2*: Attitudinal factor of student control would be a significant predictor of advocacy.

*H3*: Advocacy would mediate the relationship between institutional efficacy and direct action.

*H4*: Students control is a predictor of organizational participation.

*H5*: Direct action would mediate the relationship between advocacy and organizational participation.

*H6*: Student control would mediate the relationship between institutional efficacy and advocacy.

## Method

### Participants

The study sample included 307 university students between the ages of 18 and 26 (mean = 23, SD = 0.73). The research sample consisted of males (*n* = 153) and females (*n* = 154). The gender distribution was nearly balanced among the participants. A significant proportion of the participants fell within the age range of 24 to 26 years old (43.6% of the total). Students with any physical disability were not included in the current study.

A purposive sampling approach was used. Random sampling was not used in this study due to practical limitations. The collection of data took place during the summer, a time when a large number of students were busy with internships, which caused challenges in contacting them and consequently decreased the total response rate. Therefore, purposive sampling was selected to specifically target persons who were easily available and ready to participate.

### Measures

In this study, we adapted and redesigned the Civic Engagement Model based on Nishishiba et al. (2005) research to align with our specific institutional context. The model encompasses five variables: Institutional Efficacy (IE), Student Control (SC), Advocacy (AD), Direct Action (D.AC), and Organizational Participation (OP) (Nishishiba et al., 2005).

To establish the initial item pool for the Civic Engagement Model, we generated a set of items for each variable. To ensure the items’ quality and relevance, we conducted an expert review with an Assistant Professor from the Psychology department at Sargodha University. The review aimed to identify and eliminate any redundant, inappropriate, confusing, or emotionally charged items. Items overlapping with similar constructs were revised or removed, and the expert suggested adding relevant items. This iterative process resulted in the creation of the initial draft of the Civic Engagement Model. The study employed the following instruments in its assessment.

#### Institutional Efficacy Scale

The Institutional Efficacy Scale (IES) consists of four items (e.g., If the institution makes an effort, it has the capability to address various issues.) This self-report measure assesses attitudes toward institutional efficacy. Participants rate each item on a 5-point Likert scale, ranging from 1 (strongly disagree) to 5 (strongly agree). However, the scale’s reliability is relatively low with a Cronbach alpha of 0.68.

#### Student Control Scale

The Student Control Scale (SCS) includes three items (e.g., Research students at our institution are allowed to choose their research topic and supervisors.). This self-report measure assesses student empowerment within their institution. Participants use a 5-point Likert scale to rate each item, ranging from 1 (strongly disagree) to 5 (strongly agree). The scale’s reliability is 0.60.

Cronbach’s alpha, which is used as an index of reliability is sensitive to the item count in a scale. Thus, having more items will make reliability larger (Grieger, 2021; SPSS FAQ, 2023) as more points provide a more generalized and stable view of the construct. Hence, an item with fewer items is going to have a smaller Cronbach’s alpha. For these two scales, then, having a smaller alpha is acceptable due to having fewer items.

When assessing the reliability of scales with fewer items, Cronbach’s alpha must be understood with caution. Whereas larger alpha measures (for instance, 0.70 or greater) are generally expected, it is well known that the reliability of Cronbach’s alpha is directly influenced by the number of items in scales (DeVellis, 2017). Scholars have also pointed out that relying solely on alpha can be misleading and other considerations like content validity as well as what use is intended for the scale must be considered (McNeish, 2018). Hence, for scales with fewer items, even 0.60 and 0.68 values of alpha, though not ideal, but reasonable.

#### Advocacy Scale

The Advocacy (AD) Scale consists of five items (e.g., I have brought the issues of students to the attention of the appropriate platform to help them). This scale measures participants’ engagement in advocacy activities. The Likert scale ranges from 1 (never) to 5 (always) for each item. The scale demonstrates high reliability with a Cronbach alpha of 0.91.

#### Direct Action Scale

The Direct-Action (D.AC) Scale comprises four items (e.g., I participated in the protest to resolve the issues related to the institution.). This self-report measure assesses participants’ involvement in direct action. Each item is rated on a 5-point Likert scale, ranging from 1 (never) to 5 (always). The scale’s reliability is high, with a Cronbach alpha of 0.93.

#### Organizational Participation Scale

The Organizational Participation (OP) Scale comprises four items (e.g., I participate in various conferences and workshops). This scale measures participants’ involvement in organizational activities. The response format ranges from 1 (never) to 5 (always) for each item. The scale demonstrates strong reliability, with a Cronbach alpha of 0.90.

### Procedure

First of all, the topic was approved by the Institutional Review Board of, the Department of Psychology, University of Sargodha (IRB, 499/2023). Following the acquisition of permissions, a Google Form was created, which included all scales and demographic questions to facilitate data collection.

Google Forms were used because it is practical and economical. Most students had access to the appropriate equipment, and no major concerns with accessibility or technical difficulties were identified.

Participants were contacted in different venues and they were provided with detailed information about the purpose of the study. Keeping in view the ethical considerations, their written informed consent was taken after ensuring the confidentiality of data. All queries were answered and at the end, they were thanked for their participation in the study.

### Data analysis

Following the data collecting phase, responses were gathered in an Excel sheet, coded, and imported into IBM-SPSS for further statistical analysis. The relevant statistical tests were performed. Finally, the results were analyzed, compiled, and discussed in the study report. The final model of the present study was tested through AMOS V26. The predicted relationships in the proposed models were tested through AMOS by using maximum standard likelihood estimation (MLE). Path analysis was performed and these relationships within the model were measured for the goodness of fit and significance by absolute fit indices; χ2 goodness-of-fit statistic, the Root Mean Square Error of Approximation (RMSEA), the Goodness-of-Fit Index (GFI), relative fit indices, i.e., Normed Fit Index (NFI), Incremental Fit Index (IFI), Tucker-Lewis Index (TLI), Comparative Fit Index (CFI). The model of mediation was verified with the test of mediation by using the bootstrapping method performed on 2,000 boots strap samples in AMOS.

For the evaluation of model fit indices, recommended criteria were followed; a comparative fit index (CFI), Tucker–Lewis’s index (TLI), Normed Fit Index (NFI), and Incremental Fit Index (IFI) ≥ 0.90 (Cortina et al., 2017; Grieger, 2021); standardized root mean square residual (SRMR < 0.1) (Bentler, 1990) and the root mean square error of approximation (RMSEA), where values ≤ 0.05 (Brown, 2006; Browne and Cudeck, 1993). It has been observed that RMSEA has problems with simple path analysis especially with less degree of freedom. In these models, most of the time RAMSEA wrongly shows a poor fit, even though the model fits the data well. So, in such conditions, acceptance of the model should be based on CFI and SRM (Kenny et al., 2015).

## Results

Table 1 depicts the descriptive and psychometric properties of scales used in the current research. From standard deviation scores, it can be seen that mean scores are fairly representative of their respective distributions, and that there are only marginal differences between actual and potential ranges, which indicates that a restriction of ranges is not relevant to the present study. The results demonstrate that all of the scales have good reliability > 0.60, which supports their use in the current study. There are no significant differences among skewness and kurtosis values, indicating that all scores on the various measurements used in the current study are normally distributed.

**Table 1:** Descriptive statistics and alpha reliabilities for all study variables (*N* = 307).

					Range			
Scales	Item	*M*	*SD*	α	Potential	Actual	Skewness	Kurtosis
IE	4	14.56	2.53	0.68	1–20	6–20	−0.38	0.27
SC	3	9.98	2.84	0.60	1–15	3–15	0.09	−1.12
AD	5	15.08	5.47	0.91	1–25	5–25	−0.07	−1.16
D.AC	4	11.60	4.93	0.93	1–20	4–20	−0.15	−1.28
OP	4	12.79	4.76	0.90	1–20	4–20	−0.38	−1.10

Standard error of skewness = 0.14; Standard error of kurtosis = 0.28 for all instruments. IE, institutional efficacy; SC, student control; AD, advocacy; D.AC, direct action; OP, organizational participation.

Table 2 shows institutional efficacy is significantly and positively correlated with student control (*r* = 0.45, *p* < 0.01), advocacy (*r* = 0.41, *p* < 0.01), direct action (*r* = 0.41, *p* < 0.01), and organizational participation (*r* = 0.37, *p* < 0.01). Student control shows a positive correlation with advocacy (*r* = 0.64, *p* < 0.01), direct action (*r* = 0.58, *p* < 0.01), organizational participation (*r* = 0.54, *p* < 0.01).

**Table 2:** Pearson correlation among civic responsibility and civic engagement model (*N* = 307).

Scales	IE	SC	AD	D.AC	OP
IE	*–*	0.45^**^	0.41^**^	0.41^**^	0.37^**^
SC		–	0.64^**^	0.58^**^	0.54^**^
AD			–	0.84^**^	0.75^**^
D.AC				–	0.82^**^
OP					–

IE, institutional efficacy; SC, student control; AD, advocacy; D.AC, direct action; OP, organizational participation. ^**^Correlation is significant at the 0.01 level (2-tailed).

Furthermore, advocacy demonstrates a positive correlation with direct action (*r* = 0.84, *p* < 0.01) and organizational participation (*r* = 0.75, *p* < 0.01) while Direct action is positively correlated with organizational participation (*r* = 0.82, *p* < 0.01).

Results in Table 3 present the fit indices of the structural model of the present study. The value of the chi-square test (χ*2* = 16.2, *df* = 4, *p* = 0.004) indicates that the models fit the data well. It is evident through fit indices that the model demonstrates a good fit to the data and values of (CFI = 0.98, GFI = 0.97, TLI = 0.96, IFI = 0.98, AGFI = 0.92, NFI = 0.98) are reasonable. RMSEA value of 0.10 (*p*close = 0.04, *LL* = 0.05 & *UL* = 0.15) and standardized RMR value of 0.02 also testify the fit of the proposed model. Though the RMSEA value of 0.10 is striking, it should not be the only factor used to determine whether a model is suitable for structural equation modeling. A complete assessment of model fit is becoming increasingly important in modern psychometric practice, which identifies that no one index is perfect (Kline, 2015). Even though RMSEA is sensitive to mismatch, it can be affected by variables like the size of the sample and models’ complexity, particularly in the social sciences where data variation is widespread. The RMSEA of 0.10 in this study can be taken as a deviation that, although not ideal, does not disprove the overall efficacy of the model, particularly because other fit indices, such as the CFI, show strong model fit. Moreover, RMSEA values of approximately 0.10 are not unusual, especially when establishing the validity of novel or multi-faceted constructs. For instance, White et al. (2022) found 0.087–0.10 RMSEA values when psychometrically verifying the PREM-OB Scale™, with good CFI values of 0.931–0.977, as in the present investigation. Thus, the proposed model of serial mediation is supported. The direct and indirect effects are presented in Table 4.

**Table 3:** Model fit indices (*N* = 307).

χ^2^	*Df*	*GFI*	*AGFI*	*CFI*	*NFI*	*RMSEA*	*St. RMR*
16.2	4	0.97	0.92	0.98	0.98	0.10	0.02

**Table 4:** Standardized and unstandardized path coefficients for direct and indirect effects.

Paths	Β	*B*	*S. E of B*	*LL*	*UL*	*p*
IE ➔ SC	0.44	0.50	0.05	0.38	0.62	0.001
IE ➔ AD	0.15	0.34	0.10	0.15	0.55	0.001
SC ➔ AD	0.57	1.0	0.09	0.88	1.2	0.001
AD ➔ D.AC	0.84	0.73	0.03	0.70	0.80	0.001
SC ➔ OP	0.09	0.16	0.06	0.002	0.30	0.048
D.AC ➔ OP	0.76	0.73	0.03	0.64	0.81	0.001
IE ➔ SC ➔ OP	0.23	0.45	0.07	0.34	0.60	0.001
IE ➔ AD ➔ D.AC	0.34	0.67	0.10	0.49	0.88	0.001
IE ➔ SC ➔ AD	0.03	0.61	0.09	0.20	0.36	0.001
AD ➔ D.AC ➔ OP	0.29	0.59	0.03	0.62	0.74	0.001
SC ➔ AD ➔ D.AC	0.54	0.94	0.08	0.77	1.00	0.001
IE ➔ AD ➔ D.AC ➔ OP	0.28	0.53	0.08	0.37	0.71	0.001
IE ➔ SC ➔ AD ➔ D.AC	0.24	0.47	0.07	0.34	0.62	0.001
SC ➔ AD ➔ D.AC ➔ OP	0.44	0.74	0.07	0.58	0.88	0.001
IE ➔ SC ➔ AD ➔ D.AC ➔ OP	0.19	0.37	0.06	0.25	0.50	0.001

IE, Institutional Efficacy; SE, Student-Control; AD, Advocacy; D.AC, Direct Action; OP, Organizational Participation.

**Figure 1:**
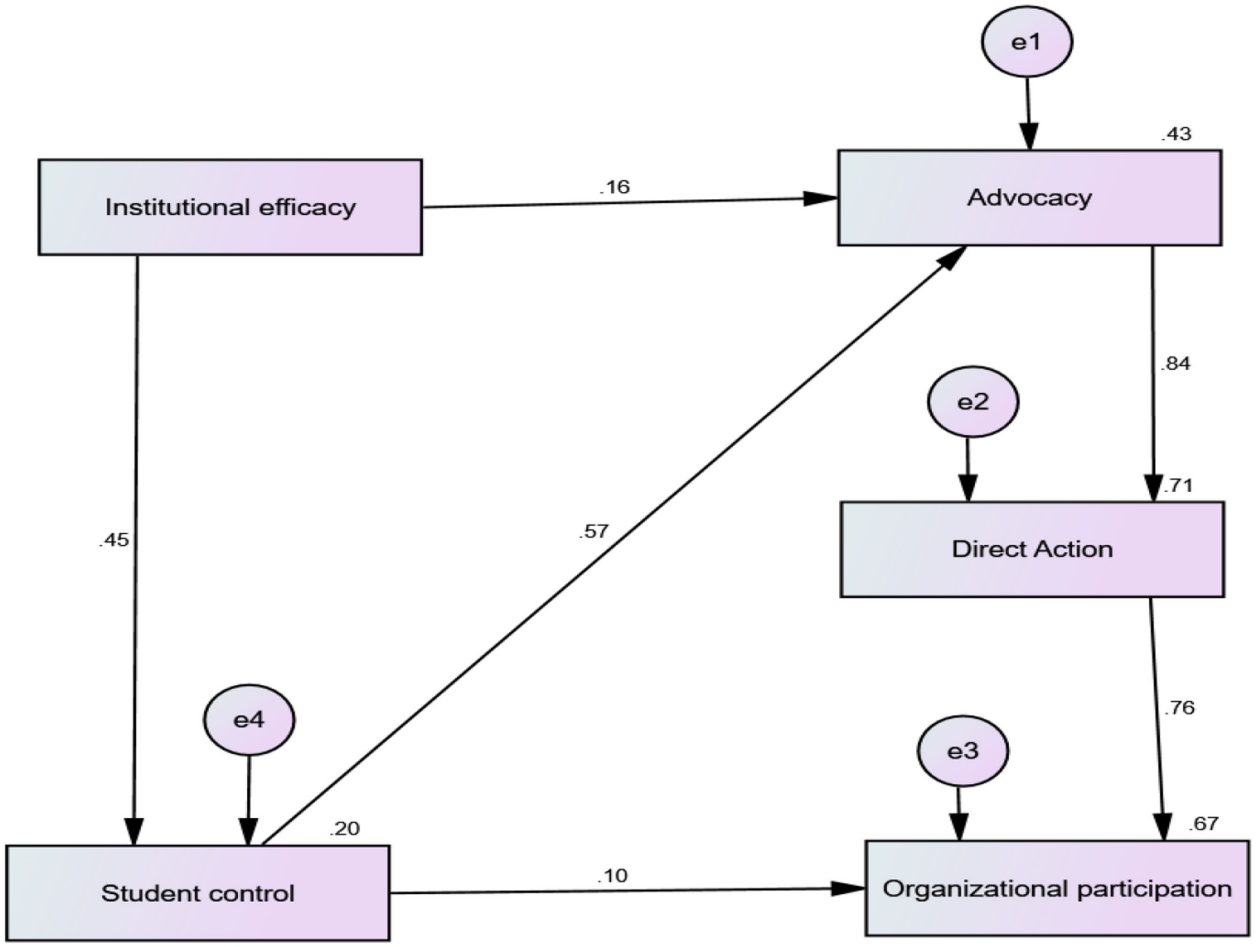
Measurement model fit of civic engagement model.

Figure 1 shows the proposed mediational model of organizational participation. Standardized coefficients are also displayed along the paths. Multiple squared correlations are represented with the rectangles of endogenous variables.

Table 4 presents standardized and unstandardized coefficients for direct and indirect effects estimated through the maximum likelihood method with 2,000 bias-corrected bootstrap samples. Results displayed a direct positive effect of institutional efficacy on self-control, which brought 19% variance in it {R^2^ = 0.19 *p* = 0.001 (*LL* = 0.11 and *UL* = 0.28)}. Results in Table 2 depicted institutional efficacy and student control as a significant positive predictor of advocacy. The indirect effect of institutional efficacy on advocacy through student control was also found significant. These direct and indirect effects explained 43% variance in advocacy {R^2^ = 0.43, *p* = 0.002 (*LL* = 0.33 and *UL* = 0.52)}. Results in Table 4 illustrated a significant positive direct effect of advocacy on direct action. The positive indirect effects of institutional efficacy on direct action through (i) advocacy and (ii) student control and advocacy were significant. The indirect positive effect of student control on direct action through advocacy was also significant. These direct and indirect effects explained 71% variance in direct action {R^2^ = 0.71, *p* = 0.001 (*LL* = 0.63 and *UL* = 0.77)}.

The results in Table 4 showed significant positive direct effects of student control and direct action on organizational participation. The positive indirect effects of institutional efficacy on organizational participation through (i) student control; (ii) advocacy and direct action and (iii) student control, advocacy, and direct action were significant. The indirect effect of advocacy on organizational participation through direct action was significant. The results in Table 4 also depicted that the positive significant impact of student control on organizational participation was serially mediated by advocacy and direct action. These direct and indirect effects explained 66% variance in organizational participation {R^2^ = 0.66 *p* = 0.003 (*LL* = 0.58 and *UL* = 0.73)}.

### Model invariance across gender

In order to compare males (*n* = 153) and females (*n* = 154) on the model of the present study, firstly, the model was freely estimated (χ*2* = 26.78, *df* = 8) and then it was estimated by constraining the paths to be equal across the two groups (χ*2* = 16.3, *df* = 4). The significant chi-square difference test indicates that the model is variant across the two groups, i.e., males and females (Δχ*2* = 10.48, *df* = 4, *p* = 0.03). This suggests the need for a follow-up test whereby each of the direct and indirect effect is tested for its invariance across the two groups. The results of these follow-up tests are presented in Table 5.

**Table 5:** Standardized and unstandardized path coefficients for direct and indirect effects across gender.

Paths	*Β _males_*	*Β _females_*	*∆β*	*p*
IE ➔ SC	0.49^***^	0.41^***^	0.08	0.42
IE ➔ AD	0.23^***^	0.10	0.13	0.000
SC ➔ AD	0.53^***^	0.58^***^	0.05	0.07
AD ➔ D.AC	0.86^***^	0.81^***^	0.05	0.09
SC ➔ OP	0.06	0.15^*^	0.09	0.04
D.AC ➔ OP	0.84^***^	0.66^***^	0.18	0.000
IE ➔ SC ➔ OP	0.26^***^	0.22^***^	0.04	0.53
IE ➔ AD ➔ D.AC	0.43^***^	0.28^***^	0.15	0.04
IE ➔ SC ➔ AD	0.32^***^	0.26^***^	0.06	0.44
AD ➔ D.AC ➔ OP	0.76^***^	0.61^***^	0.15	0.03
SC ➔ AD ➔ D.AC	0.56^***^	0.51^***^	0.05	0.55
IE ➔ AD ➔ D.AC ➔ OP	0.38^***^	0.21^***^	0.17	0.03
IE ➔ SC ➔ AD ➔ D.AC	0.28^***^	0.22^***^	0.06	0.34
SC ➔ AD ➔ D.AC ➔ OP	0.50^***^	0.38^***^	0.12	0.03
IE ➔ SC ➔ AD ➔ D.AC ➔ OP	0.24^***^	0.16^***^	0.08	0.10

IE, Institutional Efficacy; SE, Student-Control; AD, Advocacy; D.AC, Direct Action; OP, Organizational Participation.

*p < 0.05, ***p < 0.001.

Results in Table 5 show standardized path coefficients for direct and indirect effects across gender. Testing the invariance across gender indicates that the positive direct effects of institutional efficacy on advocacy and direct action on organizational participation are stronger for males as compared to females. Table 5 also shows that the positive direct effect of student control on organizational participation is stronger for females as compared to males.

Table 5 also illustrates significant variant indirect effects across gender such as the positive indirect effect of institutional efficacy on direct action through advocacy is stronger for males as compared to females. The positive direct effect of advocacy on organizational participation is also stronger for males as compared to females. Table 5 shows that the relationship between (i) institutional efficacy and organizational participation (ii) student control and organizational participation are mediated by advocacy and direct action. Both of these mediational paths are stronger for males as compared to females. All other direct and indirect paths are invariant across gender.

## Discussion

The civic activities of university students were studied in this research, with special attention to the impact of institutional efficacy and student autonomy on different modes of participation in the Pakistani setting; as such, the present study aimed to apply the model of civic engagement described by Nishishiba et al. (2005) to the particular cultural context and to extend the knowledge of the dynamics described in the introduction, that is, how one specific culture with specific gender expectations and institutional patterns informs action at the level of the individual. The objective of this study was to test the civic engagement model proposed by Nishishiba et al. (2005) in a specific cultural setting and to enhance our understanding of the relationships between attitudes and behaviors associated with civic engagement. The findings of the structural equation model (SEM) provided support for the model that was proposed, indicating that the factors of institutional effectiveness, perception of student control, advocacy, direct action, and organizational involvement all play an important part in explaining civic engagement.

The fit indices, including the chi-square test (χ2 = 16.2, df = 4, *p* = 0.004), comparative fit index (CFI) CFI = 0.98, goodness-of-fit index (GFI = 0.97), adjusted goodness-of-fit index (AGFI = 0.92), normed fit index (NFI = 0.98), RMSEA value of 0.1 0 (p close = 0.04, LL = 0.05 & UL = 0.15), and standardized root mean residual (St. RMR = 0.02), collectively suggested that the structural model fits the data well.

The chi-square test was statistically significant, and other fit indices indicated a satisfactory fit.

The findings of our study provide insight into the interrelationships of attitude, civic engagement behaviors, and prevailing gender roles within our cultural setting. Hypotheses and objectives were the basic concepts that guided our research, and the findings provide a valuable addition to the existing literature on civic participation.

The first hypothesis proposed that institutional efficacy is a strong predictor of advocacy. Our findings support this hypothesis. The standardized path coefficient (*β*) of 0.44 between institutional efficacy (IE) and advocacy (AD) was statistically significant, suggesting a positive link. This finding is consistent with the prior model (Ohmer et al., 2022), which found that people who believe communities are effective are more likely to advocate for them. The statistically significant positive link between institutional efficacy (IE) and advocacy (AD) emphasizes the importance of people’s perceptions of institutional efficiency in affecting their tendency to advocate. Individuals who believe strongly in the efficacy of institutions are more likely to see these structures as capable of bringing about beneficial change. This increased confidence is likely to drive people to actively express their support, complaints, or recommendations through advocacy channels. Essentially, a positive relationship between institutional efficacy and advocacy supports the notion that confidence in the success of institutions acts as a potent catalyst, motivating people to actively participate in attempts to promote or improve the functioning of these entities.

The hypothesis also proposed that institutional efficacy predicts direct action (D.AC). The results indicate a significant positive standardized path coefficient (β) of 0.84 from IE to D.AC, supporting the hypothesis. However, there is no clear relationship between institutional efficacy and direct action. Advocacy serves as a link between institutional effectiveness and direct action. This means that those who believe institutions are effective are more likely to advocate for civic engagement, which leads to take direct action in civic participation. Advocacy, defined as actively supporting and arguing for a cause or idea, is primarily accomplished through peaceful tactics such as discourse and the promotion of a specific viewpoint (The American Heritage, 2022). However, when peaceful measures appear insufficient, individuals or groups may resort to direct action, such as strikes, violence, or protests, to achieve their goals (Direct action, 2024; LinkedIn, 2024). The relationship between advocacy and direct action is the potential development from peaceful advocacy efforts to more assertive actions when the need for change becomes urgent or conventional discussion fails to achieve the intended results. While advocacy is often linked with peaceful approaches, it can operate as a precursor or catalyst for the implementation of direct-action measures in specific situations. So, if a person believes in the institution’s success, he will be inspired to advocate for civic concerns, leading him to take direct action to become civically involved. This finding suggests that those who believe institutions are effective are far more likely to transform their ideas into concrete and proactive civic engagement acts.

The first hypothesis lastly proposed that institutional efficacy (IE) predicts organizational engagement. The study’s findings confirm this hypothesis Standardized path coefficients reveal that persons who believe in institutional efficacy are more likely to join groups, which is mediated by factors such as advocacy and direct action. The association between institutional efficacy, advocacy, direct action, and organizational engagement suggests that faith in institutional efficacy may encourage people to advocate for and participate in civic life through direct action. Organizational participation draws individuals together around a common goal or cause, fostering collaborative efforts to effect positive change in the community and its local administration (Longley, 2022; Zencity, 2023). These findings show how belief in the effectiveness of institutions might influence civic participation and community development.

The second hypothesis stated that the attitudinal component of student control (SC) predicts advocacy (AD). The study found a significant positive path coefficient (*β*) of 0.57 between SC and AD, supporting the hypothesis. This shows that students who feel in control are more likely to engage in civic participation through advocacy. Importantly, these findings are consistent with and support the validity of the original model (Nishishiba et al., 2005) being investigated. In our study, student control refers to how students view institutions’ responses to their ideas, demands, and suggestions. When individuals believe their thoughts and suggestions are being heard, they experience a sense of control, which motivates them to participate in advocacy. This emphasizes the importance of an individual’s sense of control in shaping civic engagement habits, especially through advocacy. The study’s congruence with the established model strengthens the findings, validating the hypothesis that a perceived sense of control is critical in motivating active civic engagement.

According to the study’s fourth hypothesis, students who feel a sense of control (SC) are more willing to participate in organizational activities. The findings corroborate this hypothesis, suggesting that when people perceive their opinions and ideas are valued and considered, they are more likely to join different organizations. This highlights the significance of perceived control in shaping civic engagement practices and active participation in student organizations. The study’s conformity with the established model by Nishishiba et al. (2005) adds to the strength of the findings, underlining that a felt sense of control is a crucial motivator of active civic involvement.

The civic participation paradigm of Nishishiba et al. (2005) is broadly applicable beyond Western cultures, as this study confirms its fundamental elements. They also note Pakistani cultural differences that affect how institutional efficacy and student control predict civic involvement. In Pakistan, where political instability and institutional ineffectiveness are widespread (Ahmad, 2024; Khan et al., 2014; Memon et al., 2011). In the current study, serial mediation analysis revealed that institutional efficacy leads toward advocacy, and advocacy further leads toward direct action, ultimately resulting in organizational participation. While the original model proposes that institutional efficacy generates advocacy, in Pakistan, it may indicate that distrust in institutions’ effectiveness and a desire for reform lead to this advocacy. It indicates that when students perceive that the institution is having potential and resources to deal with the issues of students, then they feel motivated for advocacy, and it leads them to take direct actions. Advocacy is playing very important role because it is the construct that creates a link between institutional efficacy with direct actions and organizational participation. It further reveals that in Pakistani educational contexts students consider that institutions are well equipped but are not facilitating students appropriately; therefore, they feel strong motivation to resolve their issues by direct action via advocacy. This cultural factor displays that the civic engagement model is viable in Pakistan and that the perceived effectiveness of institutions may influence people’s motivation to advocate for civic causes due to the crucial need for institutional improvements.

The link between institutional efficacy, advocacy, and direct action reflects Pakistan’s typical sociopolitical condition. Dissatisfaction with institutional effectiveness can promptly develop into protests or strikes (Schoene, 2019), which may speed up the evolution from advocacy to direct action thus, highlighting the model’s relevance and cultural adaptability. Culture can affect student control and civic engagement (Pancer, 2015).

In Pakistan, students may tend to advocate and participate in organizations when they feel they have control, just like in the original concept. The civic engagement model applies to Pakistan, but it’s socio-political, economical, and cultural complications influence the relationships between institutional efficacy, student control, and civic participation. These findings show that while the model’s basic concepts apply across cultures, local and contextual circumstances may affect its presentation, demanding careful attention when using it in other cultures.

Gender disparities in this study’s structural model were found to have significant effects on civic engagement behaviors. Significant gender-specific indirect effects were observed, offering light on how various routes promote civic involvement in males and females. The results of the study show that institutional efficacy indirectly benefits direct action through advocacy more for boys than girls. This suggests that boys’ belief in institutions’ efficacy leads to direct action and advocacy, emphasizing a more complex pathway to turn institutional efficacy into civic engagement. The study also found that advocacy directly improves organizational engagement more for boys than girls. This suggests that advocating affects males’ group participation more. These gender-based differences can be explained in the light of gender roles and cultural norms. This is because males are more involved in these types of activities. In collectivistic cultures like Pakistan, males are more involved in leading tasks and communal responsibilities (Coffé and Bolzendahl, 2010; Marien et al., 2010). Due to sociopolitical contexts, males in every field of life are more confident and take bold steps. Even in academic institutions, it is perceived or directly or indirectly expected and conveyed to the students that advocacy is the characteristic of males. Women in Pakistan deal with major hindrances like gender-related prejudice, restricted access to education and jobs, and cultural practices restraining their freedoms. Low self-esteem and self-confidence resulting from this gender disparity (Li et al., 2022) might help to explain shyness and reluctance to take part in civic engagement events, including direct action, organizational participation, and advocacy. In this way, these difficulties can prevent females from fully engaging in civic life.

To grasp the possible gender disparities in civic engagement in Pakistan, it is critical to recognize the fundamental sociocultural norms that influence gender roles and opportunities. While the study of Nishishiba et al. (2005) exposes gender differences in civic engagement in the United States, its findings demand more research into how these dynamics emerge in our cultural settings. In Pakistan, traditional patriarchal systems commonly give men a more prominent position in public spheres, including politics and community issues. As a result, men may have better and much more access to civic engagement resources, networks, and platforms. Besides, differences in education, economic potential, and freedom of travel can hinder women’s participation in civic activities (Ali et al., 2022; Ammar, 2023; Ejaz and Ara, 2011).

Analyzing civic engagement requires culturally informed consideration, and specifically so when comparing contexts such as the USA and Pakistan. Civic engagement has developed in the democratic context of the USA through varied political, social, and community activities (Nishishiba et al., 2005). One important aspect of such engagement is the position of individual attitudes, specifically citizen control or efficacy, which demonstrates beliefs regarding the ability to influence change (Nishishiba et al., 2005). In the case of Pakistan, the nature of the civic environment is informed by prevailing cultural values that can actually hinder engagement, and these are particularly faced by women (Ali et al., 2022). These obstacles, such as gender roles and resource limitations, are the cause of differential engagement patterns (Ali et al., 2022). Thus, context-specific factors that are both enabling and disabling of civic engagement are to be determined for the complete appreciation of civic engagement across cultures.

My research’s emphasis on institutional effectiveness and student control as predictors is supported by the work of Nishishiba et al. (2005), which also examines what affects student engagement in political and social activities. My hypotheses are particularly mirrored in the extent to which efficacy and control determine the kinds of engagement activities, including advocacy, direct action, and organizational involvement, that my research examines. My work, however, recognizes the key significance of the Pakistani context in which patriarchal structures play an influential role in women’s political engagement that diverges from an entirely US-based framework.

## Conclusion

Findings of current study provided the evidence that civic engagement model is valid on Pakistani students. It demonstrates that almost similar institutional and personal factors are involved in civic engagement across cultures. This study contributed to the findings of previous studies, providing proof for various mediational factors involved in the development of civic engagement.

### Limitations and suggestions

In the current study, students from only one university were recruited for study and results cannot be appropriately generalized to other universities. Therefore, it is suggested that future researchers should test this model on a random sample taken from multiple institutions with diverse characteristics.

It is important to acknowledge that explicit contextual factors such as political, social, economic, educational, religious, and media influences might have influenced the results of this study. These factors are highly related to the study’s cultural context. For instance, political unpredictability, media descriptions of gender norms, and religious explanations of women’s engagement in civic life could all have had an indirect impact on participants’ responses. However, the study did not evaluate or account for these characteristics, leaving a gap about how these wider impacts may have influenced the results. Future researchers are encouraged to examine these contextual elements in greater depth to get a comprehensive understanding of their possible impact on results.

External validity or generalizability is one of the major limitations of this study. The outcomes of the study cannot be generalized because all participants were from Sargodha University. Generalization to other Pakistani or international universities or cultures is restricted by the sample of the study. To increase external validity, future studies should use a more diverse and demonstrative sample of students from different colleges and universities in different areas. Since age, socioeconomic status, cultural values, and educational systems diverge between areas, colleges, and universities, collecting wide-ranging data from different areas would make the results more relevant and generalizable.

### Implications

The current study provides empirical support to the civic engagement model in a specific cultural setting, providing insights into the complicated interactions between attitudinal factors and civic engagement practices. The findings advance our theoretical knowledge of civic engagement and have practical implications for increasing community involvement and advocacy. While our research gives useful information, it is not without restrictions. The cross-sectional nature of the data limits our capacity to determine causality. Future studies could use longitudinal designs to investigate temporal correlations between factors.

Our research contributes to the literature by expanding the civic engagement model to include serial mediation effects and studying these connections in a particular cultural setting. The results have practical significance for educators, politicians, and community organizers seeking to boost youth civic involvement. These practical implications may include including civic education within the curriculum. Helping student-led events include awareness campaigns, debates, and community service. Utilizing project-based learning and offering volunteer possibilities and internships with local organizations, helping them to gain practical experience and insights into civic participation. Formation of young advisory boards to empower students in decision-making processes and encouraging them to voice their opinions and influence local policies. Institutions should provide funding for regional initiatives that encourage adolescent participation in local communities so that working with companies, organizations, and government departments raise young civic participation.

Understanding the sequential nature of these attitudinal components can help guide focused initiatives that promote a sense of efficacy, student control, and, eventually, civic engagement.

## Data Availability

The raw data supporting the conclusions of this article will be made available by the authors, without undue reservation.
